# Microstructures in relation to temperature-induced aragonite-to-calcite transformation in the marine gastropod *Phorcus turbinatus*

**DOI:** 10.1371/journal.pone.0204577

**Published:** 2018-10-17

**Authors:** Stefania Milano, Gernot Nehrke

**Affiliations:** 1 Department of Human Evolution, Max Planck Institute for Evolutionary Anthropology, Leipzig, Germany; 2 Alfred-Wegener-Institut Helmholtz-Zentrum für Polar und Meeresforschung, Bremerhaven, Germany; Union College, UNITED STATES

## Abstract

Mollusk shells represent important archives for paleoclimatic studies aiming to reconstruct environmental conditions at high temporal resolution. However, the shells, made of calcium carbonate in the form of aragonite and /or calcite, can be altered through time which may undermine the suitability for any reconstruction based on geochemical proxies (i.e., stable isotopes, radiocarbon). At present, the diagenetic processes involved in this chemical and physical deterioration are still poorly understood. The present study aims to shed light on the onset and development of diagenetic alteration in the aragonitic shell of *Phorcus turbinatus*. To artificially mimic diagenesis, shells of *P*. *turbinatus* were exposed to elevated temperatures. The transformation of the mineral phase was monitored by means of Confocal Raman Microscopy whereas the structural changes were investigated using Scanning Electron Microscopy and Atomic Force Microscopy. The results indicate that the two distinct shell layers (prismatic layer and nacre) respond differently to the elevated temperatures, suggesting that the different microstructural organization and organic content may drive the onset and spread of the aragonite-to-calcite transformation. Furthermore, changes in the microstructural arrangement became visible prior to the mineralogical transition. Our results demonstrate that the specific physico-chemical characteristics of structurally different areas within the biogenic carbonates have to be taken into account when studying the phase transformation occurring during diagenesis.

## 1. Introduction

Most mollusks form shells with supportive and protective functions. These structures are generally composed of calcium carbonate (CaCO_3_), typically aragonite and/or calcite, hierarchically organized in a wide variety of shell morphologies and microstructures [1]. Most shells are constituted of two to five microstructurally different layers [2]. In some cases, different phases of calcium carbonate can coexist within the same shell [3,4]. Along with the mineral phase (95 –- 99.9 wt. %), the organic matrix represents a minor but important component of the shell [5]. It is known that this fraction influences the shell’s mechanical properties [6,7]. However, the exact function of most of the organic molecules is still controversially discussed, as some of them appear to additionally play a critical role in the biomineralization process [8–10].

Mollusks grow their shells periodically recording environmental information in a chronological order. For this reason, and the longevity of some species, shells of aquatic and terrestrial mollusks are commonly used to reconstruct past climatic conditions [11–13]. Geochemical and structural proxies can be used to reconstruct variables such as water temperature, precipitation, oceanographic dynamics and vegetation coverage i.e. [14–16]). This information is extremely valuable to infer environmental dynamics during times preceding the instrumental era.

However, the quality of the paleoenvironmental data deduced from fossil shells strongly depends on their preservation state. Diagenetic processes occurring after the death of the animal can irreversibly alter the shell material and subsequently undermine the reliability of the archive. Diagenesis is known to induce important mineralogical phase transformations such as the transition from aragonite into calcite [17]. Aragonite is a metastable CaCO_3_ polymorph at ambient temperature and atmospheric pressure. During diagenesis, aragonite is often transformed into the thermodynamically stable phase calcite [18]. This irreversible transformation also affects the shell microstructure and geochemical signatures, preventing the use of the altered fossils in paleoenvironmental reconstructions [19].

Different approaches are commonly applied to assess the state of preservation of biogenic carbonates. The most basic test consists of staining the material with Feigl’s solution, which allows the distinction between aragonite and calcite based on the coloration intensity [20]. Additionally, cathodoluminescence microscopy can be used to detect the Mn enrichment related to diagenetic alteration [21, 22]. More recently, Confocal Raman Microscopy (CRM) has been employed to identify carbonate polymorphs in association with diagenesis [23, 24].

Although it is straightforward to determine the occurrence of phase transformations with modern analytical methods, the physical and chemical processes controlling them are still poorly understood. Depending on the prevailing conditions, the phase transition from aragonite to calcite can occur as a fluid-mediated or solid-state reaction [25]. Previous works experimentally simulated the polymorphic transformation by exposing the shells to hydrothermal treatments [26–30]. These and other studies revealed that the fluid-mediated (neomorphic) transformation occurs through a dissolution-reprecipitation mechanism at the expense of aragonite and in favor of calcite [31, 32]. In contrast, the solid-state reaction of abiogenic aragonite appears to involve calcite nucleation on dislocations and boundaries of the original microstructures [33, 34]. However, observations on the solid-state transformation in biogenic materials are still extremely limited [19, 35]. In mollusk shells, the presence of organic molecules and the complex structural organization may have a significant influence on the phase transformation. For instance, these features appear to be related to the onset of phase transformations at lower temperatures with respect to abiogenic carbonates [36]. Nonetheless, despite recognizing these elemental differences, little is known about the processes that promote such phase transitions in biogenic materials.

To further understand the role of these two components (presence of organic molecules and complex hierarchical organization) in the diagenetic process, the present study investigates the mineralogical and structural alterations in the two microstructurally different layers of *Phorcus turbinatus* shells induced by dry heating. To experimentally reproduce the diagenetically-induced mineralogical transformation, elevated temperatures are applied to the studied shells. The temperatures chosen in this study are higher than the ones that can be expected during naturally-occurring diagenesis. However, to mimic a process that normally occurs on geological time spans, elevated temperatures are needed. These conditions allow to overstep the kinetic barrier that prevents spontaneous state transformations in systems characterized by metastable equilibrium [37, 38]. *In situ* non-destructive approaches such as CRM, Atomic Force Miscroscopy (AFM) and Scanning Electron Miscroscopy (SEM), together with Thermogravimetric Analysis (TGA) are used to identify the mechanisms and driving forces behind the mineralogical phase transformation of biogenic aragonite during dry heating.

## 2. Materials and methods

**Fig 1:**
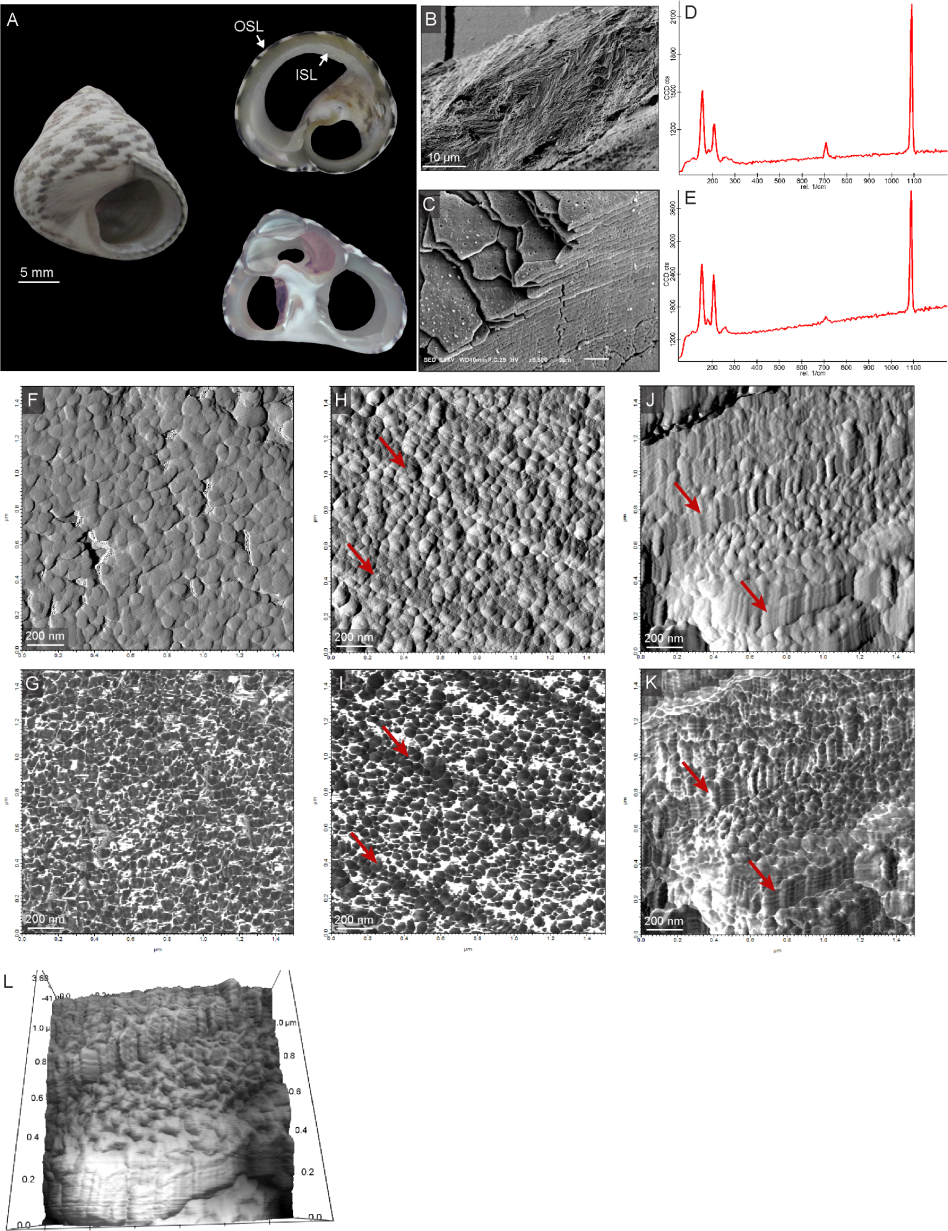
*Phorcus turbinatus* shell organization. (A) Overview of the entire shell with the transversal (upper) and longitudinal (lower) sections used in Experiment 1 and 3 and in Experiment 2, respectively. (B) SEM image of prismatic microstructure in the outer shell layer (OSL) and (C) nacre tablets in the inner shell layer (ISL). (D-E) Raman spectra of the OSL (upper) and ISL (lower) indicating that both layers share the same mineralogy (aragonite). (F) AFM amplitude and (G) phase scan of *P*. *turbinatus* OSL showing granular nanometric structures. (H+J) AFM amplitude images displaying the topographic profile of individual nacre platelets and the existence of nanometric granular subunits. (I+K) AFM phase images highlighting the inhomogeneity of the nacreous shell material. Dark grey areas identify the hard material component (aragonite) whereas lighter areas indicate the presence of softer and adhesive compounds (organic matrix). Red arrows show the location of the boundaries between platelets. (L) 3-D visualization of nacre structure.

Shells of the marine gastropod *Phorcus turbinatus*, previously known as *Osilinus turbinatus*, *Monodonta turbinata* and *Trochocochlea turbinata* [39, 40], were collected alive along the rocky shore of Sousa, Libya (32.907994°N, 22.044392°E) in 2012. After collection, the soft tissues were removed and the shells were stored at room temperature. This species is commonly found in the intertidal zones of the Mediterranean Sea and it has a conical shell characterized by two aragonitic shell layers [19, 41] (Fig 1A–1E). The two layers are easily distinguishable based on their appearance and structural organization. The Outer Shell Layer (OSL) is characterized by dark pigmented blotches alternating with white/cream portions whereas the thick Inner Shell Layer (ISL) consists of iridescent nacre. Generally, *P*. *turbinatus* deposit shell material throughout the year [42]. However, as with most of mollusk species, brief growth cessations occur periodically, which are regulated by environmental or physiological factors such as tides and stress [43]. At μm-scale, the OSL is organized in spherulitic prismatic microstructures [19] (Fig 1A and 1B). This particular arrangement consists of elongated structural units (second-order prisms) radiating with fan-like patterns from specific nucleation sites [44]. In contrast, the ISL is organized as in other species of gastropods, with 0.8–1.0 μm-thick aragonite platelets (or tablets) stacked in columns (Fig 1A–1C). A small amount of organic material composed of macromolecules, such as polysaccharides and proteins, separates each of the single carbonate units [45, 46]

### 2.1 Sample preparation

Three types of experiments were carried out with *P*. *turbinatus* shells. The duration of heating was varied between experiments, using 10 mins (Experiment 1), 12 hours (Experiment 2) and 72 hours (Experiment 3). Experiment details such as the heating device, heating rates and temperature ranges will be discussed in sections 2.2 and 2.3.

The shells used in Experiment 1 were cut across the outer whorl perpendicular to the direction of growth. Using a low-speed precision saw (Buehler Isomet 1000) two slabs of ca. 1.5 mm thickness were produced from each specimen. A total of three slabs were used in the experiment. The surface of the sections was ground using carbide papers of different grit sizes (Struers; P1200, P2400, P4000) and polished using a 0.3 μm aluminum oxide suspension applied to a silk cloth. After each grinding and polishing step, the samples were immersed for ca. 2 minutes in an ultrasonic bath containing deionized water. When dried, the slabs were cut into small fragments of 8 mm in length and 0.6 mm in thickness and glued on sapphire discs using a JB-Weld temperature-resistant resin. The shell used in Experiment 2 was prepared similarly with the exception that it was cut along the longitudinal axis (Fig 1A). One slab was used in this experiment. The four specimens used in Experiment 3 were firstly heated and then embedded in blocks of Araldite 2020 resin. Three shells (3.1, 3.2 and 3.3) were cut across the outer whorl, ground and polished following the same protocol as the shells of Experiment 1. The fourth shell was sectioned twice to produce two parallel slabs, which were prepared following the same protocol as Experiment 1 (details are given in section 2.3).

### 2.2 Thermogravimetric analysis (TGA)

Thermogravimetric analysis was performed to complement the mineralogical and structural information derived from the three heating experiments. Powdered shell samples for TGA were obtained by drilling 8.4 ± 0.2 mg of ISL and OSL using a high-precision drill (Minimo C121; Minitor Co., Ltd) with a 1-mm cylindrical bit (Komet/Gebr. Brasseler GmbH and Co. KG) attached to a binocular microscope. In order to compare the thermal behavior of biogenic aragonite to its inorganic counterpart, 9.4 mg of pure abiogenic aragonite (in house standard) was drilled using the same procedure. The three samples were weighed into aluminum oxide crucibles (70 μl) which were then closed with a perforated lid. Using a Mettler Toledo TGA/DSC 1 equipped with an autosampler, the samples were heated from 30°C to 900°C at a constant rate of 10°C/min. Powder weight loss profiles were quantified using the software STARe Excellence Version 11 (Mettler Toledo).

### 2.3 Heating and Confocal Raman Microscopy (CRM)

The mineralogy of the shells was mapped using a WITec alpha300R (WITec GmbH, Germany) confocal Raman microscope. In Experiment 1 the heating was performed using a THMS-G600 Linkham heating stage directly attached to the CRM system. Scans of 1200×600 μm and 1200×800 μm were performed on the samples using a motorized microscope stage connected to the heating stage. Each scan on average contained 93,400 single measurements with a spatial resolution of 3 μm. The shell surfaces were excited by a 488 nm-wavelength diode laser and a WITec UHTS 300 spectrometer with 600 mm^−1^ grating and 500 nm blaze was used to collect the Raman signal at an integration time between 0.08 and 0.15 s. The shells were mapped by CRM prior to heating to characterize their unaltered mineralogical composition. Subsequently, they were heated from 25°C to 250°C at a constant rate of 10°C/min. Between 250°C to 410°C series of CRM measurements were performed at regular intervals of 10, 20 or 50°C. Once the desired temperature was reached, it was held for 10 minutes before starting the measurements (Table 1). Given that the temperature sensor of the heating stage was located underneath the sample, the offset between the recorded temperature and the temperature of the sample surface was determined in a separate experiment as follows. Particles of pure indium and zinc (ca. 1 mm in diameter) with well-known melting points (156.6 and 419.5°C, respectively) were placed on the sample surface. The melting temperature was determined by clearly visible dot melting features, and the offset was calculated to be +2.4°C for In and +8.5°C for Zn. Because this offset was smaller than the narrowest temperature interval, the difference observed was considered negligible in light of the aims of this study.

**Table 1:** Details of the heating experiments on *P*. *turbinatus* shell.

Experiment	Sample ID	Heating device	Temperature range (°C)	Temperature intervals (°C)	Heating duration
1	1.1	Heating stage	250–380	10	10 mins
1	1.2	Heating stage	250–380	10	10 mins
1	1.3	Heating stage	250–410	50 (250–350°C); 20 (350–410°C)	10 mins
2	2.1	Furnace	200–300	50	12–24 hours
3	3.1	Furnace	250	-	72 hours
3	3.2	Furnace	300	-	72 hours
3	3.3	Furnace	350	-	72 hours
3	3.4	Furnace	310	-	72 hours
3	3.5	Furnace	310	-	72 hours

Experiment 2 was performed to investigate the potential influence of heating durations on the shell thermal response. These experiments were performed using a furnace (Nabertherm P320) to obtain a good comparability with Experiment 3. The shell slab was kept at 200°C for 12 hours and then it was measured by CRM. Afterwards, it was return to the furnace for an additional 24 hours at 200°C. After the second set of CRM measurements, the sample was heated again at 250°C for an extra 12 hours and subsequently heated at 300°C for 12 hours (Table 1). On average, two CRM maps (one in the ISL and one in the OSL) of 100×100 μm containing 10,000 point measurements were performed.

**Fig 2:**
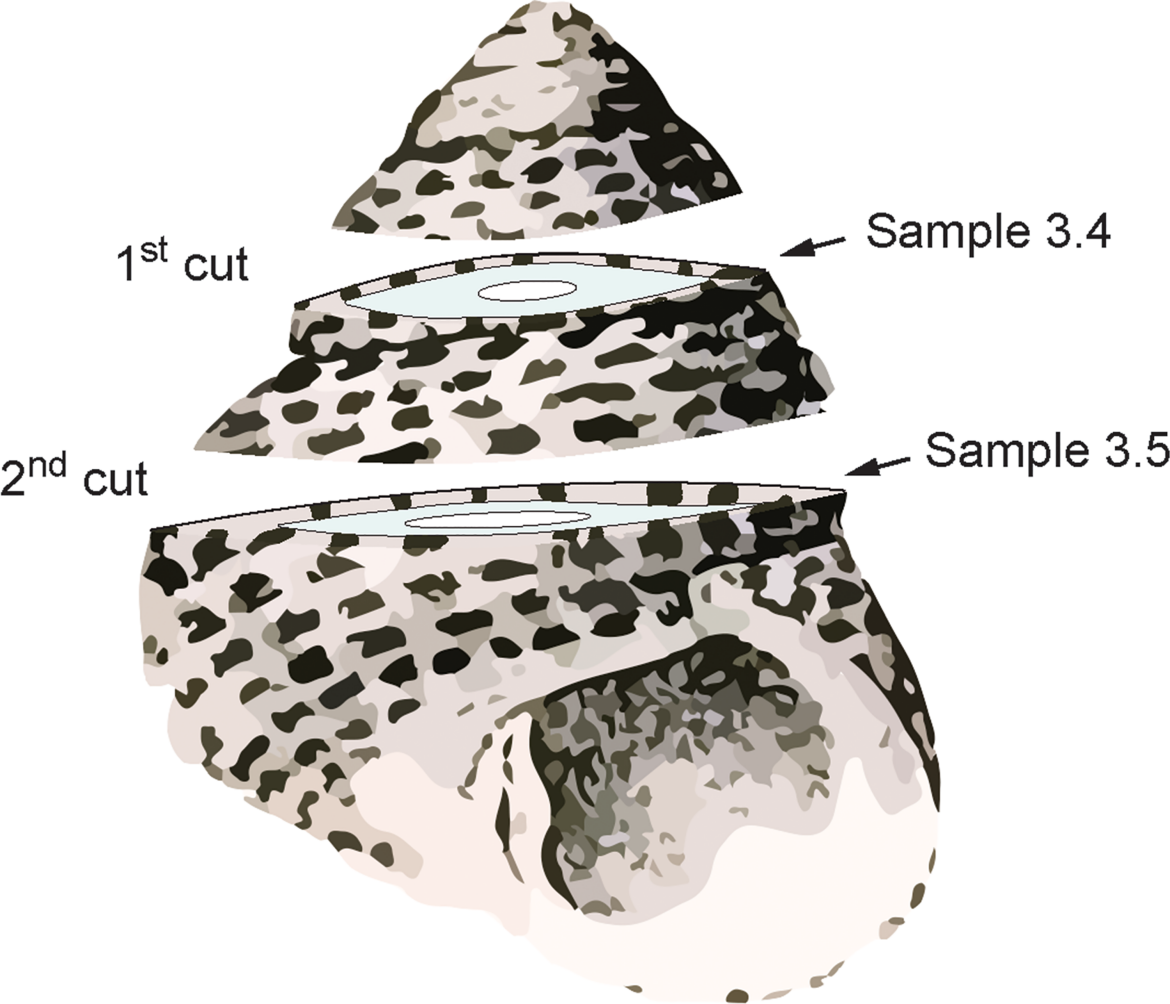
Sketch showing the preparation of samples 3.4 and 3.5. The first section was made before heating the shell. The surface (sample 3.4) was exposed to 310°C for 72 hours. After heating, the second cut was made, disclosing sample 3.5 which was not directly exposed during heating.

In Experiment 3, three shells (3.1, 3.2 and 3.3) were heated in the furnace at 250, 300 and 350°C, respectively, for 72 hours (Table 1). Each shell was mapped by CRM using the same settings as in Experiment 2. In order to test the potential variability induced by heating the whole shell (bulk) instead of a section, a direct comparison was carried out by heating one additional specimen in the furnace at 310°C for 72 hours. In this case, the tip of the shell was cut, the exposed surface was ground and polished and used as reference for the section (sample 3.4; Fig 2). After heating, the shell was cut parallel to the first cut and a second surface was prepared for the analysis. This surface was not directly exposed during heating, and therefore it was considered as reference for the bulk material (sample 3.5; Fig 2).

### 2.4 Atomic Force Microscopy (AFM) and Scanning Electron Microscopy (SEM)

In addition to the CRM, each step of the heating process of sample 1.3 was monitored using an AFM system integrated with the Raman microscope. The AFM images characterized the shell surface with nanometric resolution. The measurements were conducted in AC mode (tapping mode) using an Olympus OMCL-AC160TS cantilever with a silicon tetrahedral tip. When tracking the surface, the amplitude of the tip oscillation changes following the topography of the shell. For this reason, amplitude images are commonly used to map the roughness of materials at nm-scale. In addition, the tip phase signal is particularly sensitive to variations in the material composition, particularly in terms of viscoelasticity [47, 48]. Soft materials tend to delay the tip oscillation because they are more adhesive. As a result, AFM phase images are used to identify inhomogeneity in material properties. In this study, AFM scans of 3×3 μm and 1.5×1.5 μm were acquired in the ISL and OSL.

In Experiment 2 and 3, the shell surfaces were mapped using a MFP-3D AFM (Asylum Research) in AC mode equipped with an Olympus OMCL-AC200TS cantilever with a silicon tetrahedral tip. The ISL and OSL were characterized by scans of 3×3 μm and 1.5×1.5 μm.

In order to detect potential changes in the shell structures at μm-scale, analyses with SEM were performed. Selected shells were observed using a JSM IT100 SEM (JEOL) with 2 to 5 kV accelerating voltage.

## 3. Results

### 3.1 Shell organization at nm-scale

AFM scans of *P*. *turbinatus* reveal that both OSL prisms and nacre tablets are composed of similar basic subunits characterized by a granular morphology and a diameter of 50–70 nm (Fig 1F–1K). The AFM phase images display a clear contrast between individual granules and the area directly surrounding them (Fig 1G, 1I and 1K). According to the literature i.e. [49], the dark grey areas represent individual mineral grains enclosed in an organic sheet (white areas) which separates them from neighboring granules. As shown in Fig 1I, there is evidence of a reduced amount of organic matrix surrounding the granules at the platelet boundaries. Furthermore, these granules, when observed from an inclined angle, appear to be ordered in rods with a well-defined vertical arrangement (Fig 1J–1M).

### 3.2 Thermogravimetric analysis of powdered aragonite

**Fig 3:**
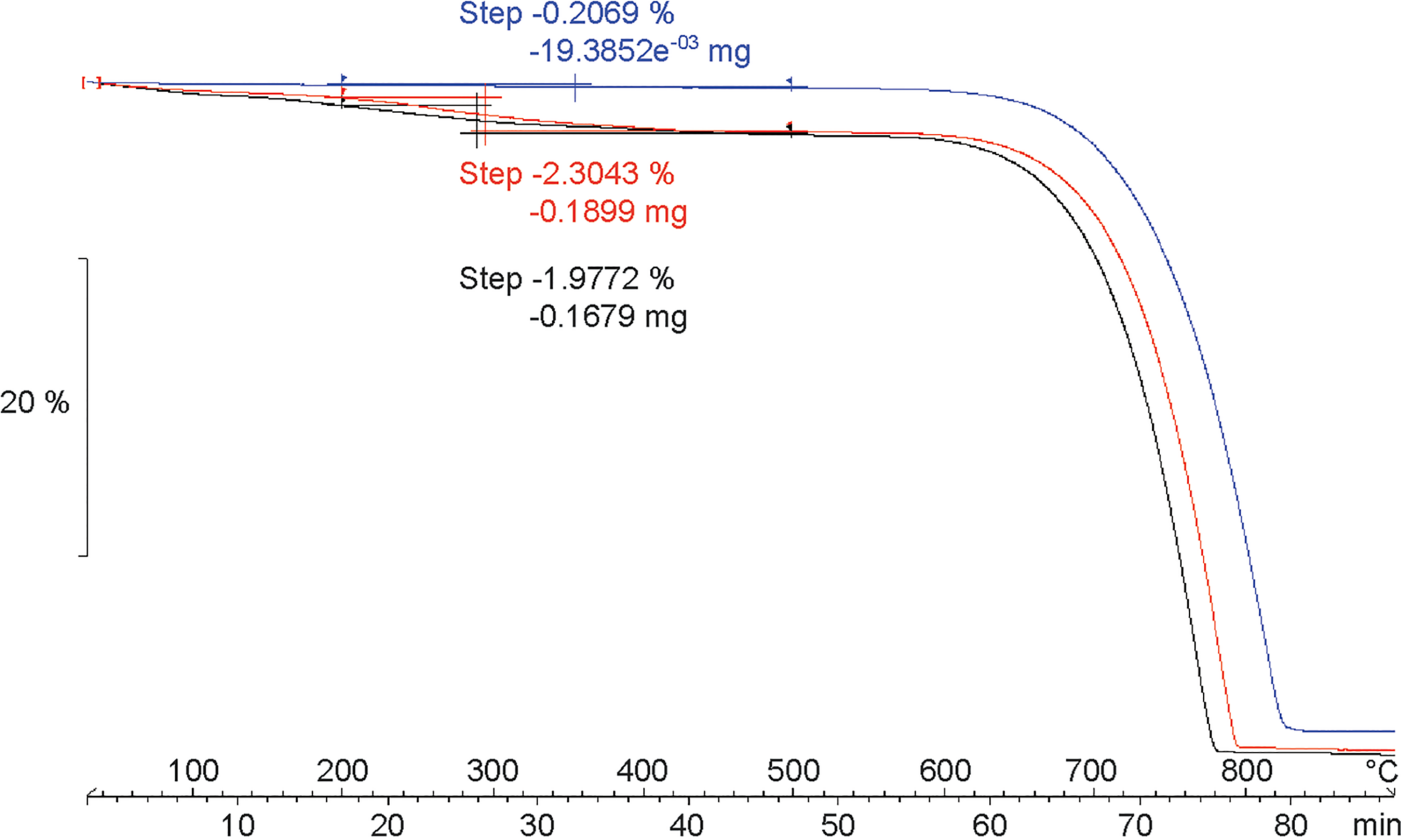
TGA thermal curve of *P*. *turbinatus* OSL (black line), nacre (red line) and abiogenic aragonite powder (blue line). For each curve, it is shown the sample weight loss (horizontal step) between 200 and 500°C.

The TGA thermal curve indicates that the major weight loss in both abiogenic aragonite and shell powder starts at around 650°C (Fig 3). However, a different thermal behavior between the two materials occurs between 200 and 500°C. The mass loss of the abiogenic aragonite in this temperature range equals 0.21%, or 0.02 mg (Fig 3). A greater loss is recorded for powdered shell. Within the same temperature range (200–500°C), the loss in the OSL is about ten-fold higher than in the abiogenic aragonite, with a decrease in mass of 1.98% or 0.17 mg. Similarly, the mass loss in the ISL is 2.30% or 0.19 mg (Fig 3).

### 3.3 Mineralogical transformation after short thermal exposure

**Fig 4:**
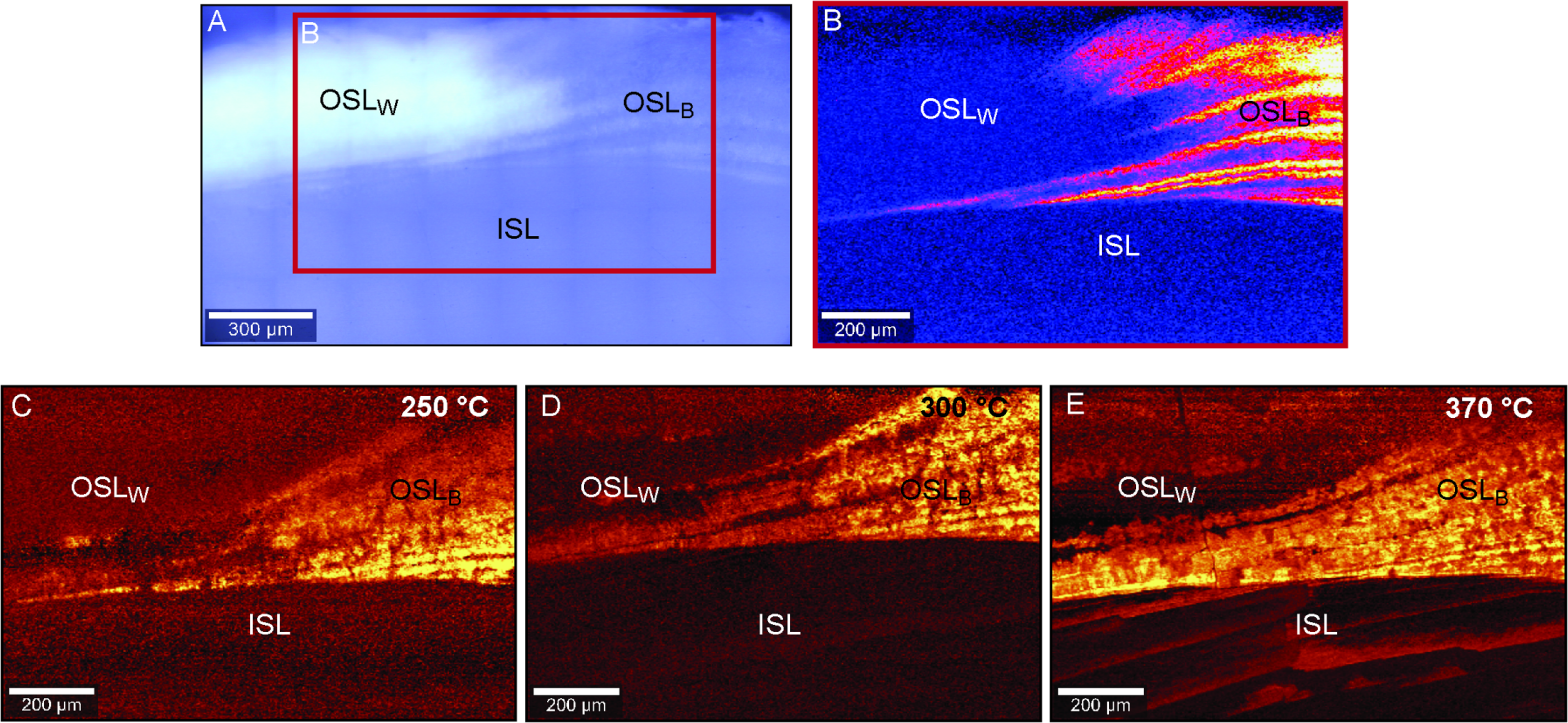
Shell pigment distribution and mineralogical response to heating. (A) Confocal microscope image of an unheated shell section (sample 1.2). The red box (B) indicates the shell portion analyzed with CRM. (B) CRM map showing the heterogeneous distribution of the pigments in the OSL based on the presence/absence of the CRM *R*_*4*_ polyene peak. In the OSL_W_ and ISL (dark blue) polyenes are absent whereas they are abundant in the OSL_B_ (pink/yellow). (C-E) CRM maps of the distribution of the different mineralogical phases in the same specimen (sample 1.2) after exposure to elevated temperatures. Dark areas are shell portions formed of aragonite, whereas bright areas indicate the presence of calcite. OSL_B_ = black portion of outer shell layer; OSL_W_ = white portion of OSL; ISL = inner shell layer (nacre).

The exposure to a short thermal treatment (Experiment 1) induces a heterogeneous mineralogical transformation in the shell (Fig 4). Although the onset of the transformation occurs at slightly different absolute temperatures among the studied specimens, they all share an identical overall trend (Table 2). In all cases, the black portion of the OSL (OSL_B_) is the first area in which the mineralogical transformation starts. It begins at 260 ± 10°C and it is completed by 327 ± 25°C (Fig 4). This is the only shell portion enriched in polyenes (Fig 4B). Presence/absence of polyenes was determined based on presence/absence of the *R*_*4*_ peak in the CRM spectrum centered at 1466 cm^-1^(with a full width at half maximum of 300 cm^-1^). This peak relates to the stretching vibrations of the polyene C = C double bonds [50, 51]. The second shell portion altered by the aragonite-calcite transformation is the white part of the OSL (OSL_W_; Fig 4). Here, the first visible signs of calcite appear at 285 ± 15°C, and the layer is fully transformed at 363 ± 15°C. The nacre (ISL) is the last portion to transform, starting at 373 ± 15°C and terminating at 390 ± 17°C. The transformation onset occurs with an offset of 25 ± 7°C between OSL_B_ and OSL_W_. The full conversion of the OSL_W_ occurs at temperatures 37 ± 14°C higher than the full conversion of the OSL_B_. Similarly, the nacre starts to transform at temperatures 113 ± 7°C higher than the OSL_B_, and it becomes fully calcitic at temperatures 63 ± 11°C higher than what is required for the full transformation of the OSL.

**Table 2:** Progression of the aragonite-calcite transformation in shell sections of Experiment 1 (short thermal exposure). OSL_B_ = black portion of outer shell layer; OSL_W_ = white portion of OSL; ISL = inner shell layer (nacre).

Sample ID	Transformation start (°C)			Transformation end (°C)		
	OSL_B_	OSL_W_	ISL	OSL_B_	OSL_W_	ISL
1.1	270	285	370	330	380	380
1.2	260	270	350	300	360	380
1.3	250	300	400	350	350	410

Likewise, the rate at which the transformation occurs differs among the three shell portions. In the OSL_B,_ calcite is first detected around 260°C, but only after 67 ± 31°C, at 350°C, the layer is fully converted. Similarly, in the OSL_W_ aragonite and calcite coexist for a 78 ± 25°C interval. However, the transformation occurs more rapidly in the ISL, where on average it takes only 17 ± 12°C before being completed.

### 3.4 Phase transformation after medium and long thermal exposure

In Experiment 2, the shell is heated at 50°C intervals from 200 to 300°C without undergoing any phase transformation. The polymorph of OSL_B,_ OSL_W_ and ISL remains aragonite (Table 3).

**Table 3:** Mineralogical composition of shells exposed to medium and long thermal exposure (Experiment 2 and 3).

Temperature (°C)	Experiment 2 (12 hours)			Experiment 3 (72 hours)		
	OSL_B_	OSL_W_	ISL	OSL_B_	OSL_W_	ISL
200	Aragonite	Aragonite	Aragonite	-	-	-
250	Aragonite	Aragonite	Aragonite	Aragonite	Aragonite	Aragonite
300	Aragonite	Aragonite	Aragonite	Partly calcite	Aragonite	Aragonite
310 (section)	-	-	-	Calcite	Calcite	Aragonite
310 (bulk)	-	-	-	Calcite	Partly calcite	Aragonite
350	-	-	-	Calcite	Calcite	Calcite

Experiment 3 (Table 3) shows that at 250°C the whole shell is still aragonitic (sample 3.1). At 300°C (sample 3.2), the OSL_B_ is partly calcitic, whereas the OSL_W_ is almost fully aragonitic and ISL remains unchanged (aragonite). The transformation is completed at 350°C (sample 3.3). At 310°C, the surface directly exposed to heat (sample 3.4) is formed of aragonite in the ISL and of calcite in the OSL. Similarly, the ISL of the bulk sample (3.5) is still aragonitic, whereas the OSL is only partially transformed. For instance, the OSL_B_ is completely calcitic, but aragonite still persists in the OSL_W_ along with the newly-transformed calcite.

### 3.5 Shell microstructural and nanostructural organization after heat exposure

**Fig 5:**
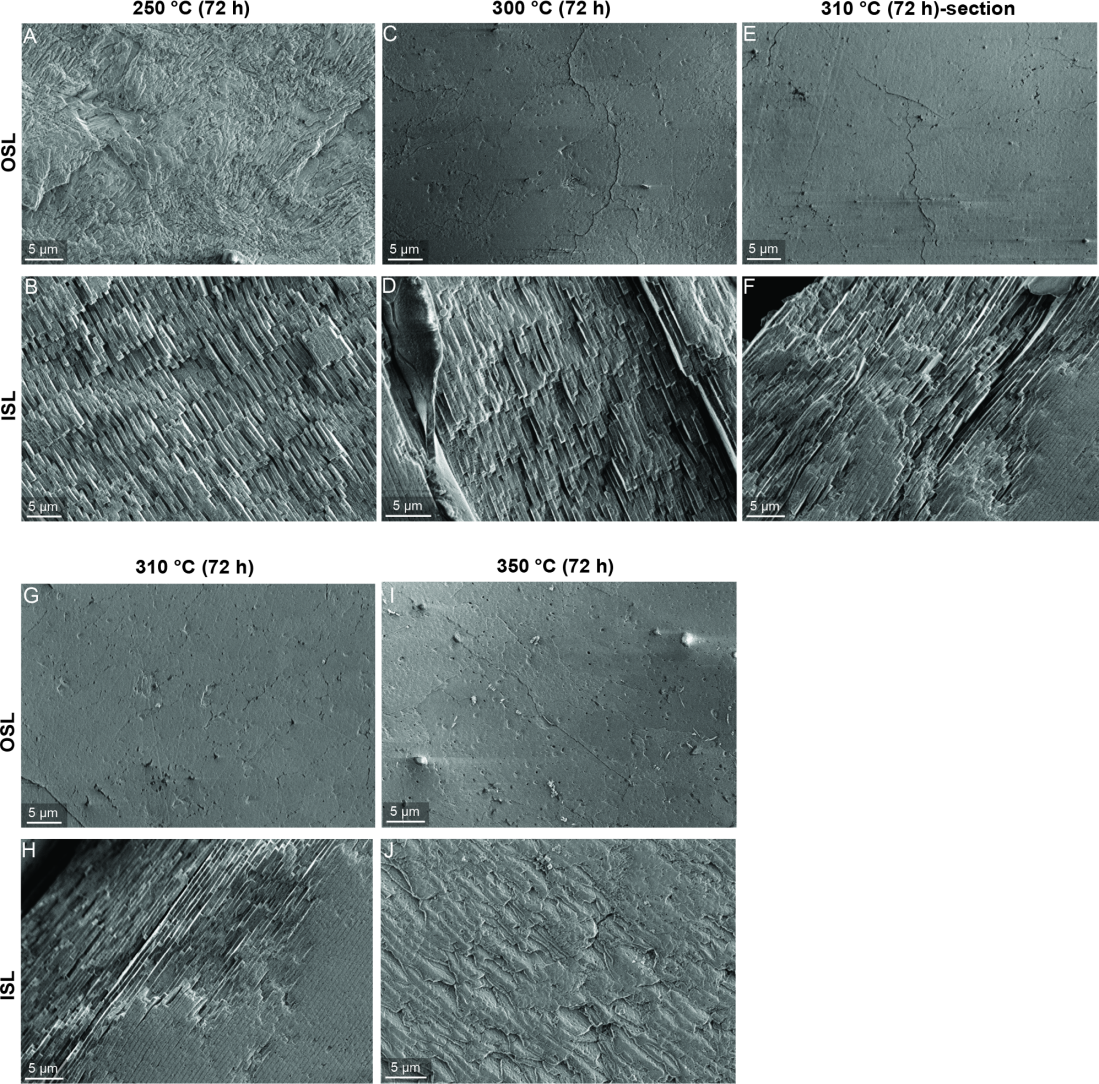
SEM images of *P*. *turbinatus* microstructures after heating (Experiment 3). Images in the first row refer to the OSL whereas the second row refers to the ISL. (E-F) Microstructures of the section directly exposed to 310°C. (G-H) Microstructures of the bulk shell heated at same temperature.

Shell architecture at μm-scale experiences important changes after being exposed to heat. As indicated by the SEM images of the Experiment 3 (Fig 5), a temperature of 250°C is associated with microstructures that closely resemble those of the unheated shells. The prismatic structures of the OSL are well defined, allowing the identification of the second-order prisms as elongated units arranged in a fan-wise design (Fig 5A). Similarly, the tablets in the nacre keep their typical geometry (Fig 5B). At 300°C, the prismatic microstructures show a major alteration, with the prisms fused with each other, contributing to a homogenous appearance of the shell layer interrupted only by narrow cracks (Fig 5C). In contrast, the tablets of the ISL show only minor alterations (i.e., pores on the tablet surface), whereas the overall organization remains unchanged (Fig 5D). Both samples heated at 310°C (section and bulk) present similar microstructures. The OSLs are characterized by the same appearance as the OSL in the previous treatment (Fig 5E–5G). Likewise, no changes can be detected in the nacre tablets (Fig 5F–5H). At 350°C, the OSL remains homogenously structured (Fig 5I), whereas the nacre displays a major alteration. The edges of the nacre tablets become irregular and distorted and, porosity increases. In some cases, the single platelets start to fuse together into larger biomineral units (Fig 5J).

**Fig 6:**
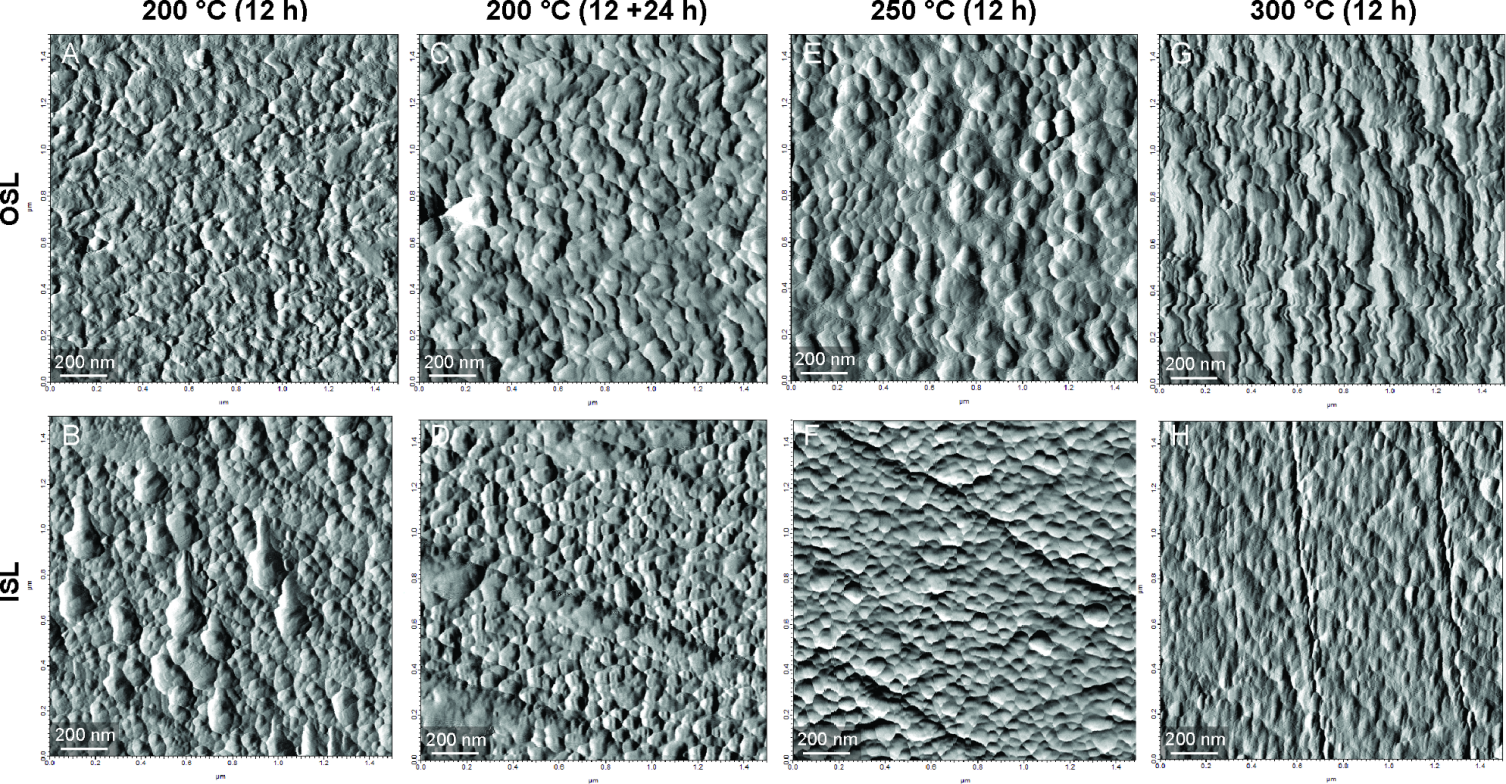
AFM scans of the nanostructures after heating (Experiment 2). Images in the first row refer to the OSL, whereas the second row represents the ISL.

Similarly, the nanostructural organization of the prisms and nacre tablets changes in response to heating. In Experiment 2, at 200°C irregular-shaped subunits begin to form in the OSL (Fig 6A). In the nacreous layer, biomineral units are larger than usual (Fig 6B). After heating the shell for another 24 hours at 200°C, the OSL seems to assume a more regular and granular organization, and the large granules of the ISL disappear (Fig 6C and 6D). At 250°C, the nanostructures are very similar to the previous treatment (Fig 6E and 6F). However, at 300°C the two layers present altered subunits (Fig 6G and 6H). In both cases, the granules lose their roundness and definition making the distinction between individual units particularly challenging. The observed changes in the nanometric structures occur in the aragonitic shell before any mineralogical transformation.

**Fig 7:**
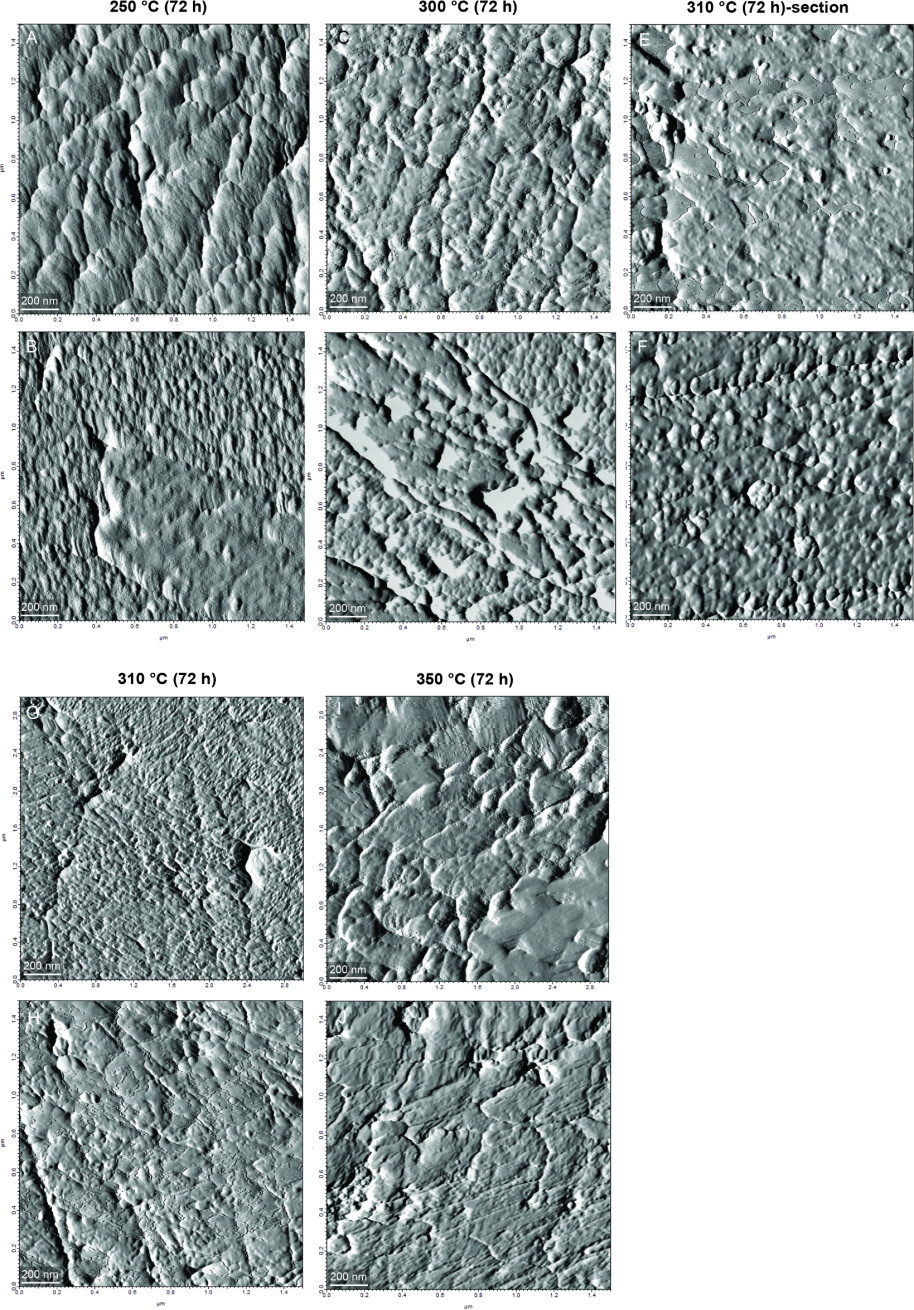
AFM scans of the nanostructures after long thermal exposure (Experiment 3). Images of the first row refer to the OSL, whereas the second row represents the ISL.

In Experiment 3, the lowest temperature (250°C) is already associated with an alteration of the typical morphology of the nm-sized granules. In the OSL, individual granules lose their definition and, when recognizable, they appear more elongated than in the unheated material (Fig 7A). In the ISL, the subunits are partially visible and rounded (Fig 7B). At 300°C, the individual granules are not identifiable in either of the two shell layers, with the exception of a few limited areas in the nacre (Fig 7C and 7D). Furthermore, large pores appear on the nacre tablet surface (Fig 7D). The section heated at 310°C shows some alterations. The OSL appears more homogenous with almost no organization at nm-scale, whereas the nacre looks slightly more structured (Fig 7E and 7F). However, the bulk specimen is more similar to the 300°C sample. There are remains of nanostructures in the OSL, whereas the nacre shows large clustered structures (Fig 7G and 7H). At 350°C, the biominerals of both layers are completely fused to form irregular and larger agglomerates (Fig 7I and 7J).

## 4. Discussion

### 4.1 Hierarchical organization of the shell of *P*. *turbinatus* at the μm- and nm-scale

According to AFM scans, the smallest building blocks of the shell of *P*. *turbinatus* are nanogranules. This arrangement is shared among the two shell layers, despite their different architecture at micrometer scale. The results are in good agreement with previous observations. For instance, strikingly similar nanometric granules were observed among numerous bivalve and gastropod species, irrespective to their shell mineralogy and organization [52–54]. Such uniformity at nm-scale compared to the large variety of structures at the micrometer scale seems to suggest that the different arrangements of the μm-structures may not result exclusively from the crystallographic disposition of nm-sized units, as proposed by the “nonclassical crystallization” models [55].

Among the different microstructures analyzed by AFM, considerable attention was given to the nacre. Previous studies showed that, as for the other types of microstructures, nacre platelets are formed by similar nm-scale granules in species studied to date [56–58]. Furthermore, by means of previous SEM and AFM analyses, nanogranules were recognized as spatially organized forming vermiculations [59]. As reported by these studies, nacre platelets presented nanometric asperities outgrowing on their free surfaces and penetrating into the interlamellar organic matrix [46, 60, 61]. At the boundaries, where two platelets are juxtaposed, their respective asperities adjoin one other. This may explain the feature of the densely-packed granules observed in *P*. *turbinatus* nacre platelet boundaries (Fig 1H–1K).

### 4.2 Thermal behavior of abiogenic and biogenic aragonite

The aragonite-calcite polymorphic transformation is an endothermic reaction during which organic molecules are decomposed and water is released. The observed major weight loss starting around 650°C is consistent with the major release of CO_2_ and subsequent aragonite decomposition into CaO [35]. At lower temperatures, the thermal profiles show a minor and more gradual weight loss. In the temperature range between 200 and 500°C, the thermal behaviors of abiogenic and biogenic aragonite are significantly different. The abiogenic aragonite curve remains virtually flat compared to the shell powdered samples. This is due to the absence of organic compounds that lower the temperature of CaCO_3_ decomposition [62]. In addition, weight loss of the shell is larger than in the abiogenic mineral, amounting to ca. 2 wt%. These results are comparable with previous works that recorded a similar weight loss, from 2 wt% up to 5 wt%, within the same temperature range [63–65]. In these studies, the nacre was the only shell portion analyzed. Discrepant results are related to different organic and water content among different mollusk species [66]. Furthermore, our results indicate that the weight loss can also differ among different shell layers. For instance, the loss of the ISL is slightly higher (0.32 wt%) than that of the OSL. Weight loss under 200°C is commonly attributed dehydration [36, 66]. Around 200°C, the first CO_2_ emissions appear, becoming increasingly more important between 300 and 500°C [62]. The released CO_2_ originates from the decomposition of the organic matrix [67]. Therefore, based on the TGA results, we can infer that the organic phase in the OSL and ISL of *P*. *turbinatus* accounts for 1.98 and 2.30% of the total shell weight, respectively. Higher organic content in the nacre might be related to the high abundance of interlamellar membranes compared to the organic sheets in the prismatic layer.

### 4.3 Mineralogical and structural transformation

The mineralogical transformation in mollusk shells has been investigated in numerous works i.e. [27, 35, 36]. In most of the cases, the process was induced by hydrothermal treatment. Under these circumstances, the conversion from aragonite into calcite occurs through a two-step mechanism. The aragonite is firstly dissolved into a local thin fluid from which the new and more stable phase is re-precipitated [33, 68, 69].

In the case of solid-state transformation, the calcite domains heterogeneously nucleate at the original aragonite grain boundaries and lattice dislocations [33, 34]. In inorganic compounds, defects in the aragonite lattice, including the appearance of nanopores, result from dehydration during heating [70]. In biogenic carbonates, beside the release of water, the degradation of the organic molecules induces strain relaxation and lattice distortion [71]. In good agreement with the previous observations, SEM and AFM scans of the present study show alteration in the structure at both micrometer and nanometer scale. At 300 and 350°C, respectively, the OSL and nacre start to display drastic microstructural changes attributable to lattice relaxation such as nanoporosity and loss of geometrical definition [68]. The nanostructures, instead, show an earlier change near 250 and 300°C in the OSL and ISL, respectively. Interestingly, all the deviations from the original arrangement occur before the mineralogical transformation. The results support the hypothesis according to which the degradation of the organic component, visible in the TGA profiles and starting around 200°C, influences the distortion of the carbonate lattice. As a result, the disordered structures induce the growth of new calcite crystals [71].

Our observations indicate that the major structural changes of the shell occur prior the mineralogical transition. Even though some minor differences can be detected, there is a striking similarity between calcite and distorted aragonite structures, supporting the importance of topotaxy in solid-state transformations that was previously observed by [34, 72]. In fact, this process involves minor atomic displacements and leads to the formation of calcite granules with a similar orientation and morphology of their aragonite precursors [34].

Previous observations on *P*. *turbinatus* suggested a heterogeneous mineralogical response to heat. Our previous results indicated that the transformation of the OSL occurs close to 300°C, whereas the nacre transforms at ca. 500°C [19]. Results herein are in good agreement with these data. Likewise, they contribute to a more detailed picture with a further distinction within the same shell layer. In fact, the black portion of the OSL is more prone to transformation than the white portion. The considerable asynchronous nature of the process emphasizes the material heterogeneity. As demonstrated, the nacre contains a larger than expected amount of organic matrix, which would be expected to lower the temperature of the conversion onset. However, the ISL is the last part to transform. In addition, the two prismatic portions of the OSL, with presumably the same quantity of inter- and intracrystalline organic matrix, do not transform simultaneously. These results suggest that the diagenetic mineralogical alteration strongly depends on the amount of and, more importantly, on the composition of the organic phase [66]. For instance, a correlation between the concentration of sulfur in the shell organic matrix and the CaCO_3_ lattice distortion has previously been reported by [73]. This observation supports the primary role of the composition of the organic phase for the structural and mineralogical transformation. Sulfur is present in the shell mainly in form of organic sulfate, and its concentration varies among the different microstructures and shell layers [74–75]. For instance, the sulfur concentration in the nacre is significantly lower than that of prismatic microstructures [66]. This enhanced chemical diversity between the shell layers can potentially explain the different thermal behavior of the OSL and ISL. The scarcity of sulfur might promote a delay in the nacre tablet lattice relaxation and aragonite-calcite transformation compared to the OSL. Furthermore, the polyenes located in the black portion of the OSL might be particularly sensitive to degradation triggering the early mineralogical conversion compared to the white portion of the OSL.

Aside from the onset of the transformation, the two shell layers differ in the rate at which the aragonite is converted into calcite. This discrepancy can be explained by the substantial difference in the arrangement of the two types of microstructures. The nucleation of calcite occurs preferably at the heterogeneities of the aragonite crystal [72–76]. The structures of the OSL, organized in small prisms with elevated orientation variability, potentially offer more heterogeneous surfaces suitable for a rapid nucleation of calcite followed by a slower growth [72]. In fact, as observed in the present study, transformation of the OSL starts early and takes long for completion, with gradual expansion of the calcite portions. In the ISL, however, the transformation is significantly faster possibly due to a faster growth of the calcite granules.

### 4.4 Methodological remarks

Despite the interest in the diagenesis of carbonate shells, little attention has been given to the structural complexity of these materials. Most of the studies used powdered samples obtained by crushing or drilling the whole shell without any distinction between the different shell layers [77, 78]. Besides having numerous varieties of microstructures, different layers are characterized by different organic and inorganic (trace elements) contents [79, 80]. Notably, the organic matrix plays an important role in the transformation process [81]. According to this basic knowledge and the results presented herein, the thermal response of the carbonates is highly inhomogeneous among shell layers. Therefore, analysis of the whole shell may introduce errors due to the mixture of the thermal response of different shell portions. Instead, the approach used in the present study, is suggested as a suitable alternative for a detailed interpretation of the diagenetic processes. Furthermore, all methods applied herein (except TGA) are non-destructive, thereby preserving the integrity of the specimens that can potentially be used for further structural and geochemical analyses.

Our results indicate that the heating duration plays only a secondary role in the mineralogical transformation. For instance, after the short (10 mins) and long (72 hours) heat exposure, the transition starts at temperatures lower than 300°C. An exception is represented by the shell of Experiment 2 heated for 12 hours at 300°C, which remained entirely aragonitic. The only distinguishable difference related to the exposure time is observed in the nacre transformation. After 72 hours, it transforms into calcite at lower temperatures than after 10 mins. These results indicate that the influence of time can be small but it has potential to become significant when considering experiments longer than 72 hours.

Furthermore, our study reveals that the thermal behavior can be different between bulk and sectioned specimens. In the case of sections, direct exposure of the shell surface to heat seems to induce a slightly faster mineralogical phase transformation. The water and organics originally contained in the shell can easily degrade and be released from the surface. However, when the shell section is not directly exposed to heat, the release rate decreases, possibly due to physical impediments during the process. This difference has to be taken into account in future studies, especially when focusing on the determination of absolute temperatures of the aragonite-calcite transformation.

Data of the present study are not sufficient to evaluate differences associated with the use of the large furnace and the use of the small heating stage connected to the CRM. Further analyses are needed to investigate the potential influence of the volume of heated space on the thermal reactions due to, for example, air flow and temperature stability. Certainly, the possibility of real-time measurements together with the high-precision temperature control are major advantages favoring the CRM heating plate. However, for prolonged experiments (> 48 hours), the use of furnaces may be preferred.

## 5. Conclusions

The thermally-induced dry diagenetic alteration of *P*. *turbinatus* shell occurs between ca. 250 and 400°C. Initially, organics are degraded and water and CO_2_ are released inducing distortion of the aragonite crystal lattice. These major structural changes provide the prerequisite for the nucleation and growth of calcite crystals. The newly formed calcitic structures closely resemble the ones of the original phase, indicating the topotactic nature of the solid-state aragonite-to-calcite transformation process. Even though the same mechanism is maintained, this process develops in the absence of uniformity across the shell. The transformation starts at first in the black portion of the OSL, expanding to the white portion of the OSL and finally to the ISL (nacre). Such heterogeneous response is likely related to the organic content of the different shell portions. Similarly, the transformation rate differs among the OSL (slow) and ISL (fast) according to the arrangement of the structures controlling the growth of the calcite crystals.

Due to the complex response of the shell to diagenesis, the structural / chemical / mineralogical analysis of shell fragments requires utmost care. To obtain robust and reproducible data, consistency is a fundamental requirement in the selection of the shell portions for any structural or geochemical analysis.

## References

[1] Carter JG, Clark GRI. Classification and phylogenetic significance of molluscan shell microstructure. In: Broadhead TW, editor. Mollusks, Notes for a Short Course. 1985. pp. 50–71.

[2] Marin F, Roy N Le, Marie B. The formation and mineralization of mollusk shell. Front Biosci S4. 2012;4: 1099–1125. 10.2741/s321 22202112

[3] Hubbard F, McManus J, Al-Dabbas M. Environmental influences on the shell mineralogy of *Mytilus edulis*. Geo-Marine Lett. 1981;1: 267–269. 10.1007/BF02462445

[4] Nehrke G, Poigner H, Wilhelms-Dick D, Brey T, Abele D. Coexistence of three calcium carbonate polymorphs in the shell of the Antarctic clam *Laternula elliptica*. Geochemistry, Geophys Geosystems. 2012;13 10.1029/2011GC003996

[5] Lowenstam HA, Weiner S. On biomineralization. New York: Oxford Press; 1989.

[6] Dashkovskiy S, Suhr B, Tushtev K, Grathwohl G. Nacre properties in the elastic range: influence of matrix incompressibility. Comput Mater Sci. 2007;41: 96–106. 10.1016/j.commatsci.2007.03.015

[7] Merkel C, Deuschle J, Griesshaber E, Enders S, Steinhauser E, Hochleitner R, et al Mechanical properties of modern calcite- (*Mergerlia truncata*) and phosphate-shelled brachiopods (*Discradisca stella* and *Lingula anatina*) determined by nanoindentation. J Struct Biol. Elsevier Inc.; 2009; 168: 396–408. 10.1016/j.jsb.2009.08.014 19729068

[8] Wheeler AP. Mechanisms of molluscan shell formation. Calcification in Biological Systems. 1992 pp. 179–216.

[9] Smith BL, Schaffer TE, Viani M, Thompson JB, Frederick N a, Kindt J, et al Molecular mechanistic origin of the toughness of natural adhesives, fibres and composites. Nature. 1999;399: 761–763. 10.1038/21607

[10] Marin F, Marie B, Hamada S Ben, Silva P, Roy N Le, Wolf SE, et al “Shellome”: proteins involved in mollusc shell biomineralization–diversity, functions. Recent Adv Pearl Res. 2013; 149–166. Available: http://hal.archives-ouvertes.fr/hal-00793668/

[11] Jones DS. Sclerochronology: reading the record of the molluscan shell. Am Sci. 1983;71: 384–391.

[12] Schöne BR, Oschmann W, Rössler J, Freyre Castro AD, Houk SD, Kröncke I, et al North Atlantic Oscillation dynamics recorded in shells of a long-lived bivalve mollusk. Geology. 2003;31: 1037–1040. 10.1130/G20013.1

[13] Stevens RE, Metcalfe SE, Leng MJ, Lamb AL, Sloane HJ, Naranjo E, et al Reconstruction of late Pleistocene climate in the Valsequillo Basin (Central Mexico) through isotopic analysis of terrestrial and freshwater snails. Palaeogeogr Palaeoclimatol Palaeoecol. Elsevier B.V.; 2012;319–320: 16–27. 10.1016/j.palaeo.2011.12.012

[14] Mutvei H, Westermark T, Dunca E, Carell B. Methods for the study of environmental changes using the structural and chemical information in molluscan shells. Bull I’lnstitut océanographique Monaco. 1994;13: 163–195.

[15] Bonadonna F, Leone G. Palaeoclimatological reconstruction using stable isotope data on continental molluscs from Valle di Castiglione, Roma, Italy. The Holocene. 1995;5: 461–469. 10.1177/095968369500500409

[16] Milano S, Schöne BR, Witbaard R. Changes of shell microstructural characteristics of *Cerastoderma edule* (Bivalvia)—A novel proxy for water temperature. Palaeogeogr Palaeoclimatol Palaeoecol. Elsevier B.V.; 2017;465: 395–406. 10.1016/j.palaeo.2015.09.051

[17] Dodd JR. Processes of conversion of aragonite to calcite with examples from the Cretaceous of Texas. J Sediment Res. 1966;36: 733–741. 10.1306/74D71555-2B21-11D7-8648000102C1865D

[18] Saylor CH. Calcite and aragonite. J Phys Chem. 1928;32: 1441–1460.

[19] Milano S, Prendergast AL, Schöne BR. Effects of cooking on mollusk shell structure and chemistry: Implications for archeology and paleoenvironmental reconstruction. J Archaeol Sci Reports. Elsevier Ltd; 2016;7: 14–26. 10.1016/j.jasrep.2016.03.045

[20] Leitmeier H, Feigl F. Eine einfache Reaction zur Unterscheidung von Calcit und Aragonit. Zeitschrift für Krist Mineral und Petrogr. 1934;45: 447–456. 10.1007/BF02943377

[21] Machel H. Cathodoluminescence in Calcite and Dolomite and Its Chemical Interpretation. Geosci Canada. 1985;12:139–47.

[22] Pagel M, Blanc P, Barbin V, Ohnenstetter D. Cathodoluminescence in Geosciences. Pagel M, Barbin V, Blanc P, Ohnenstetter D, editors. Springer-Verlag Berlin Heidelberg GmbH; 2000. 514 p.

[23] Webb GE, Price GJ, Nothdurft LD, Deer L, Rintoul L. Cryptic meteoric diagenesis in freshwater bivalves: Implications for radiocarbon dating. Geology. 2007;35: 803–806. 10.1130/G23823A.1

[24] Nouet J, Chevallard C, Farre B, Nehrke G, Campmas E, Stoetzel E, et al Limpet shells from the Aterian level 8 of El Harhoura 2 cave (Témara, Morocco): Preservation state of crossed-foliated layers. PLoS One. 2015;10: 1–26. 10.1371/journal.pone.0137162 26376294 PMC4574309

[25] Fyfe WS, Bischoff JL. The calcite-aragonite problem. Soc Econ Paleontol Mineral Spec Publ. 1965;13: 3–13. 10.2110/pec.65.07.0003

[26] Yoshioka S, Ohde S, Kitano Y, Kanamori N. Behaviour of magnesium and strontium during the transformation of coral aragonite to calcite in aquatic environments. Mar Chem. 1986;18: 35–48.

[27] Zaremba CM, Morse DE, Mann S, Hansma PK, Stucky GD. Aragonite-hydroxyapatite conversion in gastropod (Abalone) nacre. Chem Mater. 1998;10: 3813–3824.

[28] Casella LA, Griesshaber E, Yin X, Ziegler A, Mavromatis V, Müller D, et al Experimental diagenesis: Insights into aragonite to calcite transformation of *Arctica islandica* shells by hydrothermal treatment. Biogeosciences. 2016; 1–49. 10.5194/bg-2016-355

[29] Riechelmann S, Mavromatis V, Buhl D, Dietzel M, Eisenhauer A, Immenhauser A. Impact of diagenetic alteration on brachiopod shell magnesium isotope (δ26Mg) signatures: Experimental versus field data. Chem Geol. Elsevier B.V.; 2016;440: 191–206. 10.1016/j.chemgeo.2016.07.020

[30] Ritter A-C, Mavromatis V, Dietzel M, Kwiecien O, Wiethoff F, Griesshaber E, et al Exploring the impact of diagenesis on (isotope) geochemical and microstructural alteration features in biogenic aragonite. Sedimentology. 2017; 10.1111/ijlh.12426

[31] Putnis A. Mineral replacement reactions: from macroscopic observations to microscopic mechanisms. Mineral Mag. 2002;66: 689–708. 10.1180/0026461026650056

[32] Perdikouri C, Kasioptas A, Geisler T, Schmidt BC, Putnis A. Experimental study of the aragonite to calcite transition in aqueous solution. Geochim Cosmochim Acta. Elsevier Ltd; 2011;75: 6211–6224. 10.1016/j.gca.2011.07.045

[33] Kunzler RH, Goodell HG. The aragonite-calcite transformation: a problem in the kinetics of a solid-solid reaction. American Journal of Science. 1970 pp. 360–391. 10.2475/ajs.269.4.360

[34] McTigue, JW JR., Wenk H-R. Microstructures and orientation relationship in the dry-state aragonite-calcite and calcite-lime phase transformations. Am Mineral. 1985;70: 1253–1261.

[35] Yoshioka S, Kitano Y. Transformation of aragonite to calcite through heating. Geochem J. 1985;19: 245–249.

[36] Bourrat X, Francke L, Lopez E, Rousseau M, Stempfle P, Angellier M, et al Nacre biocrystal thermal behaviour. R Soc Chem. 2007;9: 1205–1208. 10.1039/b709388h

[37] Waters DJ, Lovegrove DP. Assessing the extent of disequilibrium and overstepping of prograde metamorphic reactions in metapelites from the Bushveld Complex aureole, South Africa. J Metamorph Geol. 2002;20: 135–149.

[38] Anderson GM. The entropy paradox and overstepping in metamorphic reactions. Chem Geol. Elsevier B.V.; 2014;384: 10–15. 10.1016/j.chemgeo.2014.06.020

[39] Mannino MA, Spiro BF, Thomas KD. Sampling shells for seasonality: oxygen isotope analysis on shell carbonates of the inter-tidal gastropod *Monodonta lineata* (da Costa) from populations across its modern range and from a Mesolithic site in southern Britain. J Archaeol Sci. 2003;30: 667–679. 10.1016/S0305-4403(02)00238-8

[40] Donald KM, Preston J, Williams ST, Reid DG, Winter D, Alvarez R, et al Phylogenetic relationships elucidate colonization patterns in the intertidal grazers *Osilinus Philippi*, 1847 and *Phorcus Risso*, 1826 (Gastropoda: Trochidae) in the northeastern Atlantic Ocean and Mediterranean Sea. Mol Phylogenet Evol. Elsevier Inc.; 2012;62: 35–45. 10.1016/j.ympev.2011.09.002 21945534

[41] Crothers JH. Common topshells: An introduction to the biology of *Osilinus lineatus* with notes on other species in the genus. F Stud. 2001;10: 115–160.

[42] Colonese AC, Troelstra S, Ziveri P, Martini F, Lo Vetro D, Tommasini S. Mesolithic shellfish exploitation in SW Italy: seasonal evidence from the oxygen isotopic composition of *Osilinus turbinatus* shells. J Archaeol Sci. Elsevier Ltd; 2009;36: 1935–1944. 10.1016/j.jas.2009.04.021

[43] Prendergast AL, Azzopardi M, O’Connell TC, Hunt C, Barker G, Stevens RE. Oxygen isotopes from *Phorcus* (*Osilinus*) *turbinatus* shells as a proxy for sea surface temperature in the central Mediterranean: A case study from Malta. Chem Geol. Elsevier B.V.; 2013;345: 77–86. 10.1016/j.chemgeo.2013.02.026

[44] Carter JG, Harries PJ, Malchus N, Sartori AF, Anderson LC, Bieler R, et al Illustrated glossary of the bivalvia. Treatise Online. 2012;1: 209.

[45] Checa AG, Cartwright JHE, Willinger M-G. The key role of the surface membrane in why gastropod nacre grows in towers. Proc Natl Acad Sci USA. 2009;106: 38–43. 10.1073/pnas.0808796106 19116274 PMC2629204

[46] Checa AG, Cartwright JHE, Willinger MG. Mineral bridges in nacre. J Struct Biol. 2011;176: 330–339. 10.1016/j.jsb.2011.09.011 21982842

[47] García R, Tamayo J, Paulo AS. Phase contrast and surface energy hysteresis in tapping mode scanning force microsopy. Surf Interface Anal. 1999;27: 312–316. 10.1002/(SICI)1096-9918(199905/06)27:5/6<312::AID-SIA496>3.0.CO;2-Y

[48] Bhushan B, Qi J. Phase contrast imaging of nanocomposites and molecularly thick lubricant films in magnetic media. Nanotechnology. 2003;14: 886–895.

[49] Cuif J-P, Dauphin Y, Nehrke G, Nouet J, Perez-Huerta A. Layered Growth and Crystallization in Calcareous Biominerals: Impact of Structural and Chemical Evidence on Two Major Concepts in Invertebrate Biomineralization Studies. Minerals 2012; 2:11–39.

[50] Schaffer HE, Chance RR, Silbey RJ, Knoll K, Schrock RR. Conjugation length dependence of Raman scattering in a series of linear polyenes: Implications for polyacetylene. J Chem Phys. 1991;94: 4161 10.1063/1.460649

[51] Stemmer K, Nehrke G. The distribution of polyenes in the shell of *Arctica islandica* from North Atlantic localities: a confocal Raman microscopy study. J Molluscan Stud. 2014;80: 365–370. 10.1093/mollus/eyu033

[52] Dauphin Y, Guzman N, Denis A, Cuif J, Ortlieb L. Microstructure, nanostructure and composition of the shell of *Concholepas concholepas* (Gastropoda, Muricidae). Aquat Living Resour. 2003;16: 95–103. 10.1016/S0990-7440(03)00022-6

[53] Dauphin Y. The nanostructural unity of Mollusc shells. Mineral Mag. 2008;72: 243–246. 10.1180/minmag.2008.072.1.243

[54] Wolf SE, Böhm CF, Harris J, Demmert B, Jacob DE, Mondeshki M, et al Nonclassical crystallization in vivo et in vitro (I): Process-structure-property relationships of nanogranular biominerals. J Struct Biol. Elsevier Inc.; 2016;196: 244–259. 10.1016/j.jsb.2016.07.016 27456365

[55] Cuif J-P, Dauphin Y, Sorauf JE. Biominerals and Fossils Through Time. Cambridge University Press; 2010.

[56] Li X, Chang WC, Chao YJ, Wang R, Chang M. Nanoscale structural and mechanical characterization of a natural nanocomposite material: The shell of red abalone. Nano Lett. 2004;4: 613–617. 10.1021/nl049962k

[57] Rousseau M, Lopez E, Stempflé P, Brendlé M, Franke L, Guette A, et al Multiscale structure of sheet nacre. Biomaterials. 2005;26: 6254–6262. 10.1016/j.biomaterials.2005.03.028 15907339

[58] Hovden R, Wolf SE, Holtz ME, Marin F, Muller DA, Estroff LA. Nanoscale assembly processes revealed in the nacroprismatic transition zone of *Pinna nobilis* mollusc shells. Nat Commun. 2015;6: 1–7. 10.1038/ncomms10097 26631940 PMC4686775

[59] Checa AG, Mutvei H, Osuna-Mascaró AJ, Bonarski JT, Faryna M, Berent K, et al Crystallographic control on the substructure of nacre tablets. J Struct Biol. Elsevier Inc.; 2013;183: 368–376. 10.1016/j.jsb.2013.07.014 23933391

[60] Barthelat F, Li C-M, Comi C, Espinosa HD. Mechanical properties of nacre constituents and their impact on mechanical performance. J Mater Res. 2006;21: 1977–1986. 10.1557/jmr.2006.0239

[61] Li X, Huang Z. Unveiling the formation mechanism of pseudo-single-crystal aragonite platelets in nacre. Phys Rev Lett. 2009;102 10.1103/PhysRevLett.102.075502 19257685

[62] Cuif J-P, Dauphin Y, Berthet P, Jegoudez J. Associated water and organic compounds in coral skeletons: Quantitative thermogravimetry coupled to infrared absorption spectrometry. Geochemistry, Geophys Geosystems. 2004;5 10.1029/2004GC000783

[63] Balmain J, Hannoyer B, Lopez E. Fourier transform infrared spectroscopy (FTIR) and XRD analyses of mineral and organic matrix during heating of mother of pearl (nacre) from the shell of the mollusc *Pinctada maxima*. J Biomed Mater Res. 1999;48: 749–754. 10.1002/(SICI)1097-4636(1999)48 10490692

[64] Zolotoyabko E, Pokroy B. Biomineralization of calcium carbonate: structural aspects. CrystEngComm. 2007;9: 1156–1161. 10.1039/b710974a

[65] Huang Z, Li X. Nanoscale structural and mechanical characterization of heat treated nacre. Mater Sci Eng C. Elsevier B.V.; 2009;29: 1803–1807. 10.1016/j.msec.2009.02.007

[66] Dauphin Y, Cuif JP, Salomé M, Susini J. Speciation and distribution of sulfur in a mollusk shell as revealed by in situ maps using X-ray absorption near-edge structure (XANES) spectroscopy at the S K-edge. Am Mineral. 2005;90: 1748–1758. 10.2138/am.2005.1640

[67] Dauphin Y, Cuif JP, Massard P. Persistent organic components in heated coral aragonitic skeletons-Implications for palaeoenvironmental reconstructions. Chem Geol. 2006;231: 26–37. 10.1016/j.chemgeo.2005.12.010

[68] Putnis A. Mineral replacement reactions: from macroscopic observations to microscopic mechanisms. Mineral Mag. 2002;66: 689–708.

[69] Perdikouri C, Piazolo S, Kasioptas A, Schmidt B, Putnis A. Hydrothermal replacement of aragonite by calcite: interplay between replacement, fracturing and growth. Eur J Mineral. 2013;25: 123–136. 10.1127/0935-1221/2013/0025-2261

[70] Gomez-Villalba LS, Lopez-Arce P, Alvarez de Buergo M, Fort R. Atomic defects and their relationship to aragonite-calcite transformation in portlandite nanocrystal carbonation. Cryst Growth Des. 2012;12: 4844–4852.

[71] Pokroy B, Quintana JP, Caspi EN, Berner A, Zolotoyabko E. Anisotropic lattice distortions in biogenic aragonite. Nat Mater. 2004;3: 900–902. 10.1038/nmat1263 15543151

[72] Carlson WD, Rosenfeld JL. Optical determination of topotactic aragonite-calcite growth kinetics: Metamorphic implications. J Geol. 1981;89: 615–638.

[73] Pokroy B, Fitch AN, Marin F, Kapon M, Adir N, Zolotoyabko E. Anisotropic lattice distortions in biogenic calcite induced by intra-crystalline organic molecules. J Struct Biol. 2006;155: 96–103. 10.1016/j.jsb.2006.03.008 16682231

[74] Dauphin Y, Cuif JP, Mutvei H, Denis A. Mineralogy, chemistry and ultrastructure of the external shell layer in ten species of *Haliotis* with reference to *Haliotis tuberculata* (Mollusca: Archaogastropoda). Bull Geol Institutions Univ Uppsala. 1989;15: 7–38.

[75] Dauphin Y, Cuif JP, Doucet J, Salomé M, Susini J, Willams CT. In situ chemical speciation of sulfur in calcitic biominerals and the simple prism concept. J Struct Biol. 2003;142: 272–280. 10.1016/S1047-8477(03)00054-6 12713955

[76] Koga N, Kasahara D, Kimura T. Aragonite crystal growth and solid-state aragonite-calcite transformation: A physico-geometrical relationship via thermal dehydration of included water. Cryst Growth Des. 2013;13: 2238–46.

[77] Lécuyer C. Effects of heating on the geochemistry of biogenic carbonates. Chem Geol. 1996;129: 173–183. 10.1016/0009-2541(96)00005-8

[78] Maritan L, Mazzoli C, Freestone I. Modelling changes in mollusc shell internal microstructure during firing: Implications for temperature estimation in shell-bearing pottery. Archaeometry. 2007;49: 529–541. 10.1111/j.1475-4754.2007.00318.x

[79] Dauphin Y, Ball AD, Castillo-Michel H, Chevallard C, Cuif J-P, Farre B, et al In situ distribution and characterization of the organic content of the oyster shell *Crassostrea gigas* (Mollusca, Bivalvia). Micron. 2013;44: 373–383. 10.1016/j.micron.2012.09.002 23022314

[80] Shirai K, Schöne BR, Miyaji T, Radarmacher P, Krause R a., Tanabe K. Assessment of the mechanism of elemental incorporation into bivalve shells (*Arctica islandica*) based on elemental distribution at the microstructural scale. Geochim Cosmochim Acta. 2014;126: 307–320. 10.1016/j.gca.2013.10.050

[81] Parker JE, Thompson SP, Lennie AR, Potter J, Tang CC. A study of the aragonite-calcite transformation using Raman spectroscopy, synchrotron powder diffraction and scanning electron microscopy. R Soc Chem. 2010;12: 1590–1599. 10.1039/b921487a

